# Sex-Related Differences of the Effect of Lipoproteins and Apolipoproteins on 10-Year Cardiovascular Disease Risk; Insights from the ATTICA Study (2002–2012)

**DOI:** 10.3390/molecules25071506

**Published:** 2020-03-26

**Authors:** Matina Kouvari, Demosthenes B. Panagiotakos, Christina Chrysohoou, Ekavi N. Georgousopoulou, Dimitrios Tousoulis, Christos Pitsavos

**Affiliations:** 1 Department of Nutrition and Dietetics, School of Health Science and Education, Harokopio University, 176 76 Athens, Greece; matinakouvari4@gmail.com (M.K.); ekavigeorgousopoulou@gmail.com (E.N.G.); 2 Faculty of Health, University of Canberra, Bruce ACT 2617, Australia; 3 First Cardiology Clinic, School of Medicine, University of Athens, 157 72 Athens, Greece; christina.chrysohoou@yahoo.com (C.C.); koun.dimitris@gmail.com (D.T.); mkouvari@hua.gr (C.P.); 4 School of Medicine, Sydney, The University of Notre Dame, 128-140 Broadway, Chippendale NSW 2007, Australia; 5 Medical School, Australian National University, Canberra, Canberra ACT 0200, Australia

**Keywords:** heart disease, sex, woman, lipoproteins, apolipoproteins, primary prevention

## Abstract

The sex-specific effect of lipid-related biomarkers on 10-year first fatal/non fatal cardiovascular disease (CVD) incidence was evaluated. ATTICA study was conducted during 2001–2012. *n* = 1514 men and *n* = 1528 women (>18 years) from greater Athens area, Greece were recruited. Follow-up (2011–2012) was achieved in *n* = 2020 participants. Baseline lipid profile was measured. Overall CVD event was 15.5% (*n* = 317) (19.7% in men and 11.7% in women, *p < 0.001*). High density lipoprotein cholesterol (HDL-C) and triglycerides (TAG) were independently associated with CVD in women; per 10 mg/dL HDL-C increase, hazard ratio (HR) = 0.73, 95% confidence interval (95% CI) (0.53, 1.00); and per 10 mg/dL TAG increase, HR = 1.10, 95% CI (1.00, 1.21). Apolipoprotein A1 (ApoA1) (per 10 mg/dL increase, HR = 0.90, 95% CI (0.81, 0.99)) was inversely associated with CVD in women, while a positive association with apolipoprotein B100 (ApoB100) was observed only in men (per 10 mg/dL increase, HR = 1.10, 95% CI (1.00, 1.21)). Non-HDL-C was associated with CVD in the total sample (HR = 1.10, 95% CI (1.00, 1.21)) and in women (HR = 1.10, 95% CI (1.00, 1.21)); a steep increase in HR was observed for values >185 mg/dL in the total sample and in men, while in women, a raise in CVD risk was observed from lower values (>145 mg/dL). As for non-HDL-C/HDL-C and TC/HDL-C ratios, similar trends were observed. Beyond the common cholesterol-adjusted risk scores, reclassifying total CVD risk according to other lipid markers may contribute to early CVD prevention. Biomarkers such as HDL-C, non-HDL-C, and TAG should be more closely monitored in women.

## 1. Introduction

Cardiovascular disease (CVD) is responsible for >4 million deaths in Europe each year, killing more women (i.e., 2.2 million) than men (i.e., 1.8 million) [1]. In addition to this, within the last year, a transformation in women’s risk-factor profile towards unhealthy lifestyle habits and obesity has been observed followed by an acceleration in coronary heart disease and acute myocardial infarction incidence, even in younger—pre-menopause—life stages [2]. Despite this epidemiological evidence as well as the increasing evidence that predictive risk factors differ between sexes, the vast majority of the obtained knowledge on prevention and risk prediction in the CVD spectrum is still based on studies conducted predominately in men [3].

Dyslipidemia remains a well-established CVD risk factor [4]. Numerous cohorts, Mendelian randomization studies, and randomized clinical trials have consistently demonstrated a log-linear relationship between the absolute changes in plasma low density lipoprotein cholesterol (LDL-C) and CVD risk, providing compelling evidence for a causal relation [5]. On the other side, the inverse association between plasma high density lipoprotein cholesterol (HDL-C) and risk for atherosclerotic CVD stands among the most reproducible associations in observational epidemiology, yet with weaker evidence regarding its added value as a therapeutic target [6,7]. In this regard, a sex-related heterogeneity has been previously suggested, with total cholesterol (TC) and LDL-C being stronger CVD predictors in the case of men [8,9,10] and HDL-C in the case of women [11,12]. What is more, traditional risk markers explain only a proportion of total CVD risk, principally oriented by observations in studies where women are usually underrepresented. To this issue, beyond the strong documentation and commentary on these conventional lipid biomarkers, other lipid-related factors such as non-HDL-C, TC/HDL-C, as well as their apolipoproteins are lastly investigated as potentially strong independent CVD predictors. Nevertheless, no robust conclusions exist, while their clinical use is often overlooked [4]. On the other hand, the hitherto literature related with these alternative lipid biomarkers lacks in the aforementioned need for a sex-specific orientation [13,14,15].

Thereby, the aim of the present work was to evaluate the sex-specific effect of conventional lipid-related markers (i.e., TC, LDL-C, HDL-C, triglycerides (TAG))—on the basis of existing thresholds of blood lipid concentrations—non-conventional lipid-related markers (i.e., TC/HDL-C, non-HDL-C, non-HDL-C/HDL-C), as well as their apolipoproteins on 10-year first fatal/non fatal CVD incidence, in apparently healthy men and women. Our primary research hypothesis was that, considering the biological and lifestyle discrepancies between men and women, sex-specific associations of the examined lipid-related biomarkers against CVD onset exist.

## 2. Results

The 10-year fatal/non fatal CVD event rate in the ATTICA study participants was 15.7% (*n* = 317) (19.7% (*n* = 198) in men and 11.7% (*n* = 119) in women, *p* < 0.001). Median survival time was 9.7 years in men and 9.8 years in women (*p* = 0.55). Baseline sociodemographic and clinical characteristics of participants as well as their metrics for lipid-related markers separately for men and women are summarized in Table 1.

### 2.1. Conventional Lipid-Related Biomarkers, Sex, and CVD

The findings from nested Cox regression models that evaluated the association between conventional lipid markers (i.e., TC, LDL-C, HDL-C, and TAG) and CVD incidence in free-of-CVD men and women of the ATTICA study are presented in Table 2. In the unadjusted models, a positive association was observed between all lipid-related factors and CVD in both men and women (all *p*-values *< 0.05*). However, in the age-adjusted models, several sex-specific associations were revealed. In particular, TC lost its independent aggravating effect in both men and women. In the case of men only, HDL-C in terms of continuous variable was inversely associated with CVD onset; a 10 mg/dL HDL-C increase was associated with 20% lower CVD risk within the decade. However, when adjusting for other clinical and lifestyle factors, this association remained, yet without reaching the level of significance. As for women, the age-adjusted models revealed significant associations only in the case of HDL-C and TAG. This was retained even in multi-adjusted models. Specifically, per 10 mg/dL increase in HDL-C, 10% lower CVD risk was observed, while women with HDL-C > 45 mg/dL had about 27% lower risk of developing CVD within the decade. Similarly, per 10 mg/dL rise in TAG, 10% lower CVD risk was revealed, with the risk for CVD onset being about 31% higher in women with TAG > 150 mg/dL, in the multi-adjusted model.

### 2.2. Apolipoproteins, Sex, and CVD

The association between apolipoproteins and 10-year CVD incidence was also evaluated in free-of-CVD men and women of the ATTICA study through nested Cox regression analysis, and the results are summarized in Table 3. It was revealed that, in the case of women, apolipoproteins A1 (ApoA1) was independently associated with 10-year CVD onset; particularly, per 10 mg/dL increase in ApoA1, the risk of developing CVD was 19% lower. In the case of men, besides the significant trends observed principally for apo-lipoprotein B100 (ApoB100), indicating an independent aggravating effect in age-adjusted models, this was not the case after taking into account potential confounders.

### 2.3. Alternative Lipid-Related Biomarkers, Sex, and CVD

Among the principle aims of the present work was to evaluate the effect of non-conventional lipid-related markers—highly discussed—in the recent literature in relation to primary CVD incidence. The results are presented in Table 4. In particular, the non-HDL-C variable was independently associated with 10-year CVD incidence in the total sample; a 10 mg/dL increase in this variable was associated with about a 10% rise in CVD risk. Interestingly, this effect was retained only in women. When the thresholds suggested by the European Society of Cardiology were used, a steep increase in HR was observed for values >185 mg/dL in the total sample as well as in men, while in the case of women, a significant rise in CVD risk was observed even from lower values, that is, >145 mg/dL. As for the non-HDL-C/HDL-C ratio, similar trends were observed; per 1 unit rise in this ratio, about a 13–18% increase in CVD risk was observed in the total sample as well as in the men and woman subsamples. Interestingly, when the analysis was repeated in relation to non-HDL-C/HDL-C tertiles, significant positive associations with CVD risk were observed only in the total sample and women, yet only in the case of values >3.75. In the case of TC/HDL-C, similar trends were revealed.

### 2.4. Sex-Specific Discrimination Ability of Lipid-Related Biomarkers Against CVD

The discrimination ability of multi-adjusted epidemiological models adjusted for different combinations of lipid-related biomarkers was evaluated separately for men and women, and the results are summarized in Table 5. Overall, the discrimination ability (expressed through C-index) of the examined multi-adjusted models adjusted for HDL-C and TAG or non-HDL-C or non-HDL-C/HDL-C or TC/HDL-C was better in the case of women. The correct classification rate for cases in women was more than twice as high in Models 3 and 5–7 compared with Model 2 (i.e., LDL-C adjusted model) (23.3–26.4% vs. 10.9%). On the other side, in the case of men, models adjusted for LDL-C and ApoB100/ApoA1 had a better discriminative ability against CVD. This observation was followed by the highest correct classification rates for CVD cases in models adjusted for LDL-C (i.e., Models 1 and 2).

## 3. Discussion

To the best of our knowledge, the sex-specific effect of lipid-related markers, conventional and non-conventional, on long-term CVD onset has been inadequately investigated. This work stands among the very few in the Mediterranean region that suggested different predictive ability among various lipid markers and their apolipoproteins specifying the outcomes to men and women. In particular, based on a large-scale prospective epidemiological study, it was revealed that non-HDL-C, non-HDL-C/HDL-C, and TC/HDL-C ratio had a generally higher discriminative ability against 10-year CVD onset, principally in women, compared with conventional lipid biomarkers. For men, LDL-C seemed to ameliorate the predictive ability of conventional risk prediction models even if the association was not significant. As for apolipoproteins, the ApoB100/ApoA1 ratio was a better CVD predictor in the case of men. Despite the potential limitations of the present study, the results presented here may confer to better understanding the role of lipid-related markers on CVD risk stratification, even more from the standpoint of a sex-specific approach highly suggested in the primary health care spectrum.

Dyslipidemia stands among the most important predictors of CVD. In this context, LDL-C reduction remains the primary therapeutic target in CVD with preventive and prognostic potentials. Taking into account the updates on CVD prevention and dyslipidemia management guidelines from the European Society of Cardiology, the concept “less is more” regarding LDL-C values is strongly supported [4]. Besides the high recognition of this lipid marker in therapeutic regimens to accurately prevent major cardiac episodes or achieve a better prognosis, its efficiency in the context of CVD risk prediction is still questioned. For instance, people with obesity or metabolic syndrome are assigned in the high or even very high CVD risk category, even if their lipid profile is beyond the typical one, for example, high LDL-C [16]. Indeed, such people are principally characterized by low HDL-C values, elevated levels of TAG, and a high content of small dense pro-atherogenic ApoB100 particles [16]. It is thus possible that people with a high content of elevated small dense lipoprotein particles have near normal LDL-C values owing to the discordance between the ApoB100 particle number and their cholesterol content. These people will have an underestimated risk prediction score. Hence, the high residual risk for CVD as well as the different phenotypes that exist have driven lipid research towards non-conventional surrogate lipid markers such as ApoB100, ApoA1, non-HDL-C, small dense LDL-C particles, Lp(a), and so on. Additionally, the combined assessment of two lipid markers through ratios such as TC/HDL-C and non-HDL-C/HDL-C is highly suggested as stronger CVD predictors. In this respect, much as the HDL-C hypothesis is increasingly discussed, it is important to emphasize that its value as a CVD predictor remains highly unchallenged [17]. Many prospective studies from different racial and ethnic groups have confirmed that HDL-C is a strong, consistent, and independent predictor of incident cardiac episodes [18]. On the other side, accumulating evidence suggests that non-HDL-C and ApoB100 are superior to LDL-C in predicting CVD risk, while both have been designated as secondary targets in some treatment guidelines [19]. It is thus obvious that the traditional view on the relationship between lipid biomarkers and CVD risk has changed, yet without strong evidence that non-conventional lipid biomarkers are able to confer a better predictability of CVD risk compared with more traditional ones.

To the best of our knowledge, this work stands among the very few that examined the role of conventional and non-conventional lipid biomarkers against hard CVD-related endpoints, specifying the outcomes for men and women. Focusing on the traditional lipid markers, what we revealed was that LDL-C was an important CVD predictor only in men, yet in the case of women, HDL-C seemed to contribute more to the overall CVD risk. Men have higher LDL-C levels compared with the age-matched women, till the menopause stage; because then, a steep LDL-C rise occurs, predisposing women to a CVD-risk escalation [20]. On the other side, HDL-C and triglycerides have been suggested as stronger lipid indicators in the case of women, which comes in line with findings arisen here. More specifically, HDL-C addition in SCORE risk stratification led to a modest improvement in its predictive ability for women [21]. In a Women’s Health Study, the inverse association between HDL-C and primary CVD incidence was retained across all LDL-C levels, with the exception of women with low total atherogenic particle burden [20]. What is more, the TC/HDL-C ratio has been suggested as a strong independent predictor for acute myocardial infarction in men with only a paucity of studies evaluating this ratio in women [8,13,22]. In line with previous works, the findings raised here suggested that TC/HDL-C ratio is independently associated with increased CVD risk in both sexes, yet with the association being stronger in the case of women. As for triglycerides, in a relevant meta-analysis, a stronger association of fasting triglycerides with CVD mortality in women was revealed [23].

Among the primary purposes of the present work was to evaluate the sex-specific effect of alternative lipid-related markers against hard CVD endpoints. The added value of non-HDL-C and relative derivatives such as the non-HDL-C/HDL-C ratio is increasingly suggested within the last decade, yet with inconclusive outcomes. Here, we showed that the lowest hazard for CVD was found in women and men with the lowest non-HDL-C concentrations. This is in accordance with outcomes revealed from the Multinational Cardiovascular Risk Consortium, where a continuous and linear increase for higher non-HDL-C concentrations was observed [15]. Additionally, several works suggest that the non-HDL-C/HDL-C ratio is superior to traditional lipid markers in estimating arterial stiffness and atherosclerosis, with this association being more evident in the case of women [24,25]. This comes in line with the outcomes revealed here. The exact reason for this result must be determined in future studies. Changes in the levels of both HDL-C and triglycerides found to exert stronger effects among women may partially explain this observation [26] In the INTERHEART study, dyslipidemia, defined as the ratio of (ApoB100)/(ApoA1), possessed the highest population-attributed risk in both sexes [27]. Here, we saw that ApoB100 was a stronger CVD predictor for men, and yet ApoA1 was for women. However, apolipoproteins seem to have the lowest discriminative ability against CVD in both men and women, which comes in line a meta-analysis of cohort studies implemented by the Emerging Risk Factors Collaboration [6].

### Strengths and Limitations

Several limitations should be presented for better interpretation of the observed outcomes. Specifically, only baseline measurements were taken into account for our research hypothesis; hence, misclassifications of transitions cannot be precluded owing to the extended interim periods between follow-up assessments. Additionally, the ATTICA study participants presented a generally mild dyslipidemic profile at baseline along with relatively small variability of the lipid levels among individuals; this may contribute to—unexpectedly—non-significant trends in the case of biomarkers such as TC and LDL-C; these characteristics of the ATTICA study sample may modest the strength of the observed associations between lipid indices and 10-year CVD risk.

The aforementioned limitations are compensated for with several strengths. First of all, in the present work, we evaluated the sex-based effect of not only conventional, but also non-conventional lipid-related markers on a 10-year CVD onset; to the best of our knowledge, the evidence-based data regarding this issue are inadequate. Secondly, this in one of the very few prospective studies that provided metrics for discrimination and classification parameters on this issue.

## 4. Materials and Methods

### 4.1. Study Sample

The ATTICA study is a prospective, observational cohort investigation that was initiated in 2001 [28]. At baseline (2001–2002), *n* = 3042 apparently healthy volunteers residing in the greater metropolitan Athens area, Greece agreed to participate (75% participation rate). Of the enrolled participants, *n* = 1514 (49.8%) were men (46 ± 13 years) and *n* = 1528 (50.2%) were women (45 ± 14 years). During baseline examination, a detailed clinical evaluation was performed by trained physicians; all participants were free of CVD and other chronic diseases, according to the study protocol. For the scope of the present work, we initially used the *n* = 2020 participants with complete CVD evaluation in the follow-up assessment.

### 4.2. Bioethics

The ATTICA study was approved by the Bioethics Committee of Athens Medical School. The study was carried out in accordance with the Declaration of Helsinki (1989) of the World Medical Association. All participants were informed about the study aims and procedures and provided written informed consent.

### 4.3. Lipid-Related Markers Measurements at Baseline Examination

Blood samples were collected from an antecubital vein, between 08:00 and 10:00, in a sitting position after 12 h fasting and alcohol avoidance. Serum for blood lipid measurements was prepared immediately after collection. The biochemical evaluation was carried out in a laboratory that followed the criteria of the World Health Organization Lipid Reference Laboratories. Serum total cholesterol, HDL-C, and triglycerides were measured using the chromatographic enzymic method using a Technicon automatic analyzer RA-1000 (Dade Behring, Marburg, Germany). HDL-C was determined after precipitation of the ApoB-containing lipoproteins with dextran-magnesium-chloride. LDL-C (mg/dL) was calculated using the Friedewald formula: (total cholesterol) − (HDL-C) − 1/5 × (triglycerides) (only for participants with triglycerides < 400 mg/dL). ApoB100 and apoA1 were measured by rate immunonephelometry. An internal quality control was in place for assessing the validity of cholesterol, triglycerides, and HDL-C methods. The intra- and inter-assay coefficients of variation of cholesterol levels did not exceed 9%, triglycerides 4%, and HDL-C 4%. Cut-off values of LDL-C, TAG, and non-HDL-C were defined according to the most updated guidelines for dylipidaemias [4]. In the case of HDL-C, the sex-specific cut-off values suggested in NCEP ATP III (revised) criteria for metabolic syndrome were used. For TC/HDL and non-HDL-C/HDL-C variables, participants were categorized according to the generated tertiles owing to the lack of national or European thresholds; in our sample, this categorization contributed to the best discriminative ability against the outcome of interest, that is, the 10-year CVD event.

### 4.4. Other Baseline Measurements

The assessed sociodemographic and lifestyle characteristics included age, sex, body mass index, level of adherence to Mediterranean diet, physical activity level, and smoking habits. Height was measured to the nearest 0.5 cm and weight to the nearest 100 g. Body mass index was calculated as weight (in kg) divided by squared height (in m^2^). Dietary habits were evaluated through a semi-quantitative food-frequency questionnaire (FFQ), originally developed for the European Prospective Investigation into Cancer and Nutrition study and provided by the Unit of Nutrition of Athens Medical School in its Greek version [28]. Level of adherence to Mediterranean diet was evaluated through the MedDietScore (range 0–55) [29]. Current smokers were defined as those who smoked at least one cigarette per day. Physical activity level was recorded through a translated, validated version of International Physical Activity Questionnaire.

Further details regarding the methods and measurements applied have been previously described [30,31].

### 4.5. Endpoint and Follow-Up Evaluation

During 2011–2012, the ATTICA study’s investigators performed the 10-year follow-up (median follow-up time of 8.41 years). In order to participate in the follow-up, all participants were initially appointed through telephone calls. Afterwards, the investigators of the ATTICA study (physicians, nurses, nutritionists) approached the participants and performed a detailed evaluation of their medical records. For the participants who died during the follow-up (i.e., *n* = 99), the information was achieved from their relatives, as well as death certificates. The combined endpoint studied in this work was the development of a fatal or non-fatal CVD event. A CVD event was defined as the development of the following: acute myocardial infarction, unstable angina, other identified forms of ischemia (WHO-ICD coding 410–414.9, 427.2, 427.6), heart failure of different types and chronic arrhythmias (WHO-ICD coding 400.0–404.9, 427.0–427.5, 427.9), or stroke (WHO-ICD coding 430–438).

### 4.6. Statistical Analysis

Categorical variables are presented as absolute (*n*) and relative frequencies (%). Continuous variables are presented as mean values ± standard deviation or median (interquartile range) if normality was not met. Associations between normally distributed variables and sex were evaluated through Student’s *t*-test for independent samples. Whether these variables were normally distributed was tested through P–P plot and equality of variances through Levene’s test. For non-normally distributed variables, the Mann–Whitney test was used. Associations between categorical variables and sex were tested with the chi-squared test. Hazard ratios (HRs) and their corresponding 95% confidence intervals (95% CIs) for lipid-related markers in relation to 10-year CVD event were evaluated through multivariable Cox-regression analysis in the total sample, as well as in subgroups. Proportional hazards’ assumption was graphically tested. Total or CVD case-related correct classification rate was also obtained from the aforementioned multivariate analyses. The concordance statistics, that is, C-statistics, was used to evaluate the predictive accuracy of multivariate models adjusted for various lipid markers against the 10-year CVD event. C-indexes and the corresponding 95% CIs were equal to the areas under the curve obtained from the receiver operating curve (ROC) analysis. The STATA software, version 14 (MP & Associates, Sparta, Greece) was used for all statistical analyses. Two-sided level of significance was set at *p < 0.05*.

## 5. Conclusions

**Figure 1:**
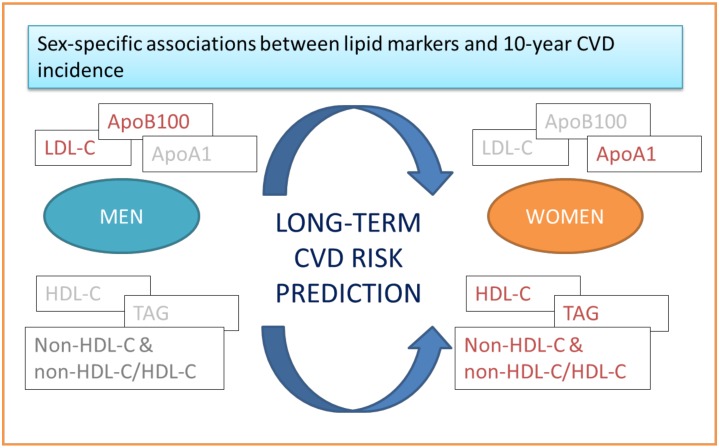
Graphical illustration of the sex-specific association of conventional and non-conventional lipid markers with long-term CVD onset based on results from the ATTICA prospective study.

While ever increasing efforts have sought to elucidate the lipid-related biomarkers that contribute more or less to the CVD risk in the primary prevention, recommendations remain to be guided with appropriate conclusive evidence, mostly from a sex-oriented approach [32]. The findings presented here partially address the literature gaps in the following key areas. Firstly, much as LDL-C is the key therapeutic target to achieve a healthier vascular system, its contribution to early CVD risk prediction seems to be questioned; here, high LDL-C levels were independently associated with increased CVD only in men. Secondly, HDL-C accompanied or not by triglycerides seemed to contribute more to women’s CVD risk compared with other conventional lipid markers. Lastly, alternative lipid-related markers, predominately non-HDL-C particles, seemed to independently increase long-term CVD, principally in women. In this regard, beyond the commonly used TC-adjusted risk scores in primary prevention spectrum, more prospective studies are demanded to investigate the incremental value of reclassifying total CVD risk according to the most updated dyslipidaemia-related evidence. Sex differences should be an indispensable part of this process (Figure 1).

## Figures and Tables

**Table 1:** Baseline sociodemographic, clinical, biochemical, and lifestyle factors of men and women from the ATTICA study, according to 10-year cardiovascular disease incidence (*n* = 2020).

**Men**	**With 10-Year CVD Event**	**Without 10-Year CVD Event**	***p*-value**
*N*	*198*	*808*	
Age, years	56 (13)	43 (12)	*<0.001*
Body mass index, kg/m^2^	28.3 (4.0)	27.1 (3.9)	*0.001*
Waist circumference, cm	101.5 (11.3)	97.0 (12.9)	*<0.001*
Current smoking, %	28	38	*<0.001*
MedDietScore, range 0–55	22.4 (6.4)	24.5 (5.2)	*<0.001*
History of hypertension, %	51	36	*<0.001*
History of diabetes mellitus, %	22	5	*<0.001*
History of hypercholesterolemia, %	58	44	*<0.001*
TC, mg/dL	206 (43)	195 (42)	*0.001*
LDL-C, mg/dL	135 (42)	125 (37)	*0.01*
HDL-C, mg/dL	44 (13)	41 (10)	*0.01*
TAG, mg/dL	182 (63)	133 (86)	*<0.001*
non-HDL-C, mg/dL	164 (44)	151 (43)	*<0.001*
TC/HDL-C	5.22 (1.81)	4.70 (1.52)	*0.001*
non-HDL-C/HDL-C	4.22 (1.81)	3.70 (1.52)	*0.001*
ApoA1, mg/dL	144 (23)	147 (25)	*0.13*
ApoB100, mg/dL	124 (27)	112 (29)	*<0.001*
Family history of cardiovascular disease, %	29	26	*<0.001*
**Women**	**With 10-Year CVD Event**	**Without 10-Year CVD Event**	**p-value**
*N*	*119*	*895*	
Age, years	59 (12)	42 (13)	*<0.001*
Body mass index, kg/m^2^	27.3 (5.1)	24.9 (4.7)	*<0.001*
Waist circumference, cm	89.7 (14.1)	82.4 (13.3)	*<0.001*
Current smoking, %	39	45	*<0.001*
MedDietScore, range 0–55	23.4 (6.4)	28.1 (6.6)	*<0.001*
History of hypertension, %	49	20	*<0.001*
History of diabetes mellitus, %	19	3	*<0.001*
History of hypercholesterolemia, %	55	36	*<0.001*
TC, mg/dL	208 (41)	189 (40)	*<0.001*
LDL-C, mg/dL	131 (36)	117 (36)	*0.08*
HDL-C, mg/dL	45 (12)	53 (14)	*0.006*
TAG, mg/dL	126 (66)	94 (54)	*<0.001*
non-HDL-C, mg/dL	156 (40)	135 (41)	*<0.001*
TC/HDL-C	4.47 (2.05)	3.75 (1.25)	*0.001*
non-HDL-C/HDL-C	3.47 (2.05)	2.75 (1.25)	*0.001*
ApoA1, mg/dL	158 (23)	163 (26)	*0.03*
ApoB100, mg/dL	108 (29)	101 (49)	*0.08*
Family history of cardiovascular disease, %	37	29	*<0.001*

Data are presented as mean ± standard deviation (SD) or median (interquartile range) if normality was not met. *p*-values were obtained using Student’s *t*-test for independent samples for the normally distributed variables (age, body mass index), Mann–Whitney test for the rest quantitative variables and chi-squared test for categorical variables. Abbreviations: apolipoprotein A1 (ApoA1); apolipoprotein B100 (ApoB100); high density lipoprotein cholesterol (HDL-C); low density lipoprotein cholesterol (LDL-C); total cholesterol (TC); triglycerides (TAG).

**Table 2:** Cox regression analysis to evaluate the association between conventional lipid markers and 10-year first fatal/non fatal cardiovascular disease incidence in apparently healthy men and women (*n* = 2020).

**Men** (*n* = 1006/*n* = 198 CVD cases)			
	**Unadjusted (Crude) Model**	**Age-Adjusted model**	**Fully Adjusted Model**
**Model for TC**			
TC, per 10 mg/dL increase	**1.10 (1.00, 1.21)**	1.00 (0.90, 1.10)	1.00 (0.90, 1.10)
TC (>200 vs. ≤200 mg/dL))	**1.75 (1.28, 2.40)**	1.24 (0.87, 1.75)	1.21 (0.85, 1.73)
**Model for LDL**			
LDL, per 10 mg/dL increase	1.00 (0.90, 1.10)	1.00 (0.90, 1.10)	1.00 (0.90, 1.10)
LDL status (>100 vs. ≤100 mg/dL)	**1.57 (1.00, 2.50)**	0.90 (0.54, 1.52)	1.10 (0.57, 2.13)
**Model for HDL**			
HDL, per 10 mg/dL increase	**0.81 (0.66, 0.90)**	**0.81 (0.66, 0.90)**	0.81 (0.66, 1.21)
HDL status (<50 vs. ≥50 mg/dL)	1.42 (0.91, 2.21)	1.44 (0.87, 2.46)	1.36 (0.81, 2.31)
**Model for TAG**			
TAG, per 10 mg/dL increase	1.10 (1.00, 1.21)	1.00 (0.90, 1.10)	1.00 (0.90, 1.10)
TAG status (>150 vs. ≤150 mg/dL)	**2.29 (1.39, 3.77)**	1.10 (0.63, 1.92)	1.60 (0.24, 1.49)
**Women** (*n* = 1014/*n* = 119 CVD cases)			
	**Unadjusted (Crude) Model**	**Age-Adjusted Model**	**Multi-Adjusted Model**
**Model for TC**			
TC, per 10 mg/dL increase	**1.10 (1.00, 1.21)**	1.00 (0.90, 1.10)	1.00 (0.90, 1.10)
TC (>200 vs. ≤200 mg/dL))	**2.19 (1.49, 3.23)**	0.96 (0.42, 1.69)	0.91 (0.58, 1.43)
**Model for LDL**			
LDL, per 10 mg/dL increase	**1.10 (1.00, 1.21)**	1.00 (0.90, 1.10)	1.00 (0.90, 1.10)
LDL status (>100 vs. ≤100 mg/dL)	**2.66 (1.50, 4.72)**	1.19 (0.63, 2.25)	2.10 (0.72, 2.57)
**Model for HDL**			
HDL, per 10 mg/dL increase	**0.73 (0.66, 0.90)**	**0.73 (0.66, 0.90)**	**0.73 (0.53, 1.00)**
HDL status (<40 vs. ≥40 mg/dL)	**1.53 (1.07, 2.17)**	**1.65 (1.12, 2.43)**	**1.44 (1.17, 2.14)**
**Model for TAG**			
TAG, per 10 mg/dL increase	**1.10 (1.00, 1.21)**	**1.10 (1.00, 1.21)**	**1.10 (1.00, 1.21)**
TAG status (>150 vs. ≤150 mg/dL)	**2.14 (1.52, 3.03)**	**1.60 (1.09, 2.34)**	**1.31 (1.01, 2.12)**

HRs and their corresponding 95% CIs were obtained through Cox regression analysis. Multi-adjusted model was adjusted for age, body mass index, current smoking, MedDietScore, hypertension, diabetes mellitus, lipid-lowering treatment, and family history of cardiovascular disease. **Bold** indicates statistically significant outcomes (*p*-value *< 0.05*). **Abbreviations:** cardiovascular disease (CVD); hazard ratio (HR); high density lipoprotein cholesterol (HDL-C); low density lipoprotein cholesterol (LDL-C); total cholesterol (TC); triglycerides (TAG); 95% confidence interval (95% CI).

**Table 3:** Cox regression analysis to evaluate the association between apolipoproteins and 10-year first fatal/non fatal cardiovascular disease incidence in apparently healthy men and women (*n* = 2020).

**Men** (*n* = 1006/*n* = 198 CVD cases)			
	**Unadjusted (Crude) Model**	**Age-Adjusted Model**	**Multi-Adjusted Model**
**Model for ApoB100**			
ApoB100, per 10 mg/dL increase	**1.21 (1.10, 1.34)**	**1.10 (1.00, 1.21)**	**1.10 (1.00, 1.21)**
**Model for ApoA1**			
ApoA1, per 10 mg/dL increase	**0.81 (0.66, 0.90)**	**0.81 (0.66, 0.90)**	0.81 (0.66, 1.21)
**Model for ApoB100/ApoA1**			
ApoB100/ApoA1, per 1 unit increase	**1.63 (1.03, 2.57)**	1.18 (0.73, 1.89)	0.93 (0.56, 1.54)
**Women** (*n* = 1014/*n* = 119 CVD cases)			
	**Unadjusted (Crude) Model**	**Age-Adjusted Model**	**Multi-Adjusted Model**
**Model for ApoB100**			
ApoB100, per 10 mg/dL increase	**1.10 (1.00, 1.21)**	1.00 (0.90, 1.10)	1.00 (0.90, 1.10)
**Model for ApoA1**			
ApoA1, per 10 mg/dL increase	**0.81 (0.66, 0.90)**	**0.90 (0.81, 0.99)**	**0.90 (0.81, 0.99)**
**Model for ApoB100/ApoA1**			
ApoB100/ApoA1, per 1 unit increase	1.40 (0.89, 2.22)	0.83 (0.34, 2.00)	0.69 (0.25, 1.88)

HRs and their corresponding 95% CIs were obtained through Cox regression analysis. Multi-adjusted model was adjusted for age, body mass index, current smoking, MedDietScore, hypertension, diabetes mellitus, lipid-lowering treatment, and family history of cardiovascular disease. **Bold** indicates statistically significant outcomes (*p-*value < 0.05). **Abbreviations:** apolipoprotein A1 (ApoA1); apolipoprotein B100 (ApoB100); cardiovascular disease (CVD); hazard ratio (HR); 95% Confidence Interval (95% CI).

**Table 4:** Cox regression analysis to evaluate the association between non-conventional lipid markers and 10-year first fatal/non fatal cardiovascular disease incidence in apparently healthy men and women (*n* = 2020).

Total (*N*/Cases)			Men (*N*/Cases)			Women (*N*/Cases)		
*2020/317*			*1006/198*			*1014/119*		
**Non-HDL-C**	**CVD incidence, %**	**HR (95% CI)**	**Non-HDL-C**	**CVD incidence, %**	**HR (95%CI)**	**Non-HDL-C**	**CVD incidence, %**	**HR (95% CI)**
per 10 mg/dL	**-**	**1.10** **(1.00, 1.21)**	per 10 mg/dL	**-**	1.00 (0.90, 1.10)	per 10 mg/dL	**-**	**1.10** **(1.00, 1.21)**
<100 mg/dL	5.3	*ref*	<100 mg/dL	7.6	*ref*	<100 mg/dL	4.2	*ref*
100–<145 mg/dL	13.3	1.18 (0.61, 2.27)	100–<145 mg/dL	18.2	2.69 (0.91, 6.50)	100–<145 mg/dL	9.4	2.32 (0.89, 5.34)
145–<185 mg/dL	18.0	1.16 (0.60, 2.24)	145–<185 mg/dL	20.4	3.12 (0.89, 4.50)	145–<185 mg/dL	14.8	**3.45** **(1.09, 7.43)**
185–<220 mg/dL	20.0	**2.10** **(1.54, 3.26)**	185–<220 mg/dL	21.6	**3.04** **(1.32, 4.44)**	185–<220 mg/dL	17.3	**3.64** **(1.10, 5.96)**
>220 mg/dL	28.9	**2.95** **(1.24, 4.32)**	>220 mg/dL	33.9	**3.14** **(1.26, 5.10)**	>220 mg/dL	20.6	**3.79** **(1.20, 6.20)**
**Non-HDL-C/HDL-C**	**CVD incidence, %**	**HR (95% CI)**	**Non-HDL-C/HDL-C**	**CVD incidence, %**	**HR (95% CI)**	**Non-HDL-C/HDL-C**	**CVD incidence, %**	**HR (95% CI)**
per 1 unit	**-**	**1.15** **(1.06, 1.26)**	per 1 unit	**-**	**1.13** **(1.02, 1.26)**	per 1 unit	**-**	**1.18** **(1.02, 1.36)**
<2.49	8.4	*ref*	<2.49	13.2	*ref*	<2.49	6.5	*ref*
2.49–3.71	15.2	1.27 (0.85, 1.90)	2.49–3.71	17.7	0.95 (0.53, 1.71)	2.49–3.71	12.4	1.25 (0.70, 2.21)
>3.71	22.0	**1.96** **(1.34, 2.86)**	>3.71	24.4	1.35 (0.78, 2.33)	>3.71	18.9	**1.72** **(1.10, 3.07)**
**TC/HDL-C**	**CVD incidence, %**	**HR (95% CI)**	**TC/HDL-C**	**CVD incidence, %**	**HR (95% CI)**	**TC/HDL-C**	**CVD incidence, %**	**HR (95% CI)**
per 1 unit	**-**	**1.15** **(1.06, 1.26)**	per 1 unit	**-**	**1.13** **(1.02, 1.26)**	per 1 unit	**-**	**1.18** **(1.02, 1.36)**
<3.49	8.4	*ref*	<3.49	13.2	*ref*	<3.49	6.5	*ref*
3.49–4.71	15.2	1.10 (0.73, 1.66)	3.49–4.71	17.7	0.95 (0.53, 1.71)	3.49–4.71	12.4	1.25 (0.70, 2.21)
>4.71	22.0	**1.54** **(1.04, 2.29)**	>4.71	23.4	1.35 (0.78, 2.33)	>4.71	18.9	**1.72** **(1.01, 3.07)**

HRs and their corresponding 95% CIs were obtained through Cox regression analysis adjusted for age, body mass index, current smoking, MedDietScore, hypertension, diabetes mellitus, lipid-lowering treatment, and family history of cardiovascular disease. **Bold** indicates statistically significant outcomes (*p*-value < 0.05). **Abbreviations:** Cardiovascular disease (CVD); hazard ratio (HR); high density lipoprotein cholesterol (HDL-C); total cholesterol (TC); 95% confidence interval (95% CI).

**Table 5:** Discrimination-ability parameters of multivariate models adjusted for different combinations of lipid markers over the 10-year first fatal/non-fatal cardiovascular disease event (*n* = 2020).

Models	Model Adjustment Description	C-Index (95% CI)	Correct Classification Rate, % (Total)	Correct Classification Rate, % (Cases)
**Model 1**	Standard model * adjusted for **conventional lipid markers^⸠^**	**Men**		
		0.772 (0.713, 0.831)	83.6	33.3
		**Women**		
		0.831 (0.777, 0.886)	89.6	19.6
**Model 2**	Standard model * adjusted for **LDL-C**	**Men**		
		0.830 (0.789, 0.872)	88.6	21.9
		**Women**		
		0.772 (0.728, 0.816)	82.8	10.9
**Model 3**	Standard model * adjusted for **HDL-C & TAG**	**Men**		
		0.784 (0.741, 0.827)	83.0	18.3
		**Women**		
		0.829 (0.795, 0.877)	89.3	24.4
**Model 4**	Standard model * adjusted for **ApoB100/ApoA1**	**Men**		
		0.833 (0.792, 0.874)	88.6	23.9
		**Women**		
		0.776 (0.734, 0.818)	82.4	13.3
**Model 5**	Standard model * adjusted for **non-HDL-C**	**Men**		
		0.769 (0.729, 0.809)	82.4	13.0
		**Women**		
		0.836 (0.790, 0.869)	89.1	23.3
**Model 6**	Standard model * adjusted for **non-HDL-C/HDL-C**	**Men**		
		0.772 (0.732, 0.812)	82.2	15.0
		**Women**		
		0.833 (0.793, 0.873)	89.1	26.2
**Model 7**	Standard model * adjusted for **TC/HDL-C**	**Men**		
		0.772 (0.732, 0.812)	82.2	15.0
		**Women**		
		0.836 (0.793, 0.873)	89.1	26.2

* Standard model was adjusted for age, body mass index, current smoking, MedDietScore, hypertension, diabetes mellitus, and family history of cardiovascular disease. ^⸠^ Conventional lipid markers examined were low density lipoprotein cholesterol, high density lipoprotein cholesterol, and triglycerides. C-index and the corresponding confidence interval were evaluated through the area under the curve obtained from the receiver operating characteristics (ROC) analysis. ROC analysis was performed using the probabilities for 10-year first fatal/non-fatal cardiovascular disease event, corresponding to each study participant, separately for men and women, calculated from Cox regression analysis using the multivariate models described. Correct classification rate was obtained from the Cox regression analysis performed using the described models, separately for men and women. **Abbreviations:** apolipoprotein A1 (ApoA1); apolipoprotein B100 (ApoB100); high density lipoprotein cholesterol (HDL-C); low density lipoprotein cholesterol (LDL-C); total cholesterol (TC); triglycerides (TAG).
